# Scientific leadership and sustainable innovation in Chinese knowledge-intensive firms: unpacking the roles of talent ecosystem and green innovation capability

**DOI:** 10.3389/fpsyg.2026.1811652

**Published:** 2026-04-24

**Authors:** Rongcheng Liang

**Affiliations:** 1 Emergency Management Training Department, Party School of Shandong Provincial Committee of the Communist Party of China (Shandong Academy of Governance), Jinan, China; 2 School of International Relations and Public Affairs, Fudan University, Shanghai, China

**Keywords:** environmental dynamism, green innovation capability, knowledge-intensive firms, scientific leadership, sustainable innovation, talent ecosystem

## Abstract

Drawing upon dynamic capabilities theory, this study investigates how scientific leadership shapes sustainable innovation through the mediating mechanisms of talent ecosystem and green innovation capability in Chinese knowledge-intensive firms. Using multi-source survey data from 287 firms across high-technology sectors, the researcher found that scientific leadership positively influences both talent ecosystem development and green innovation capability. Furthermore, talent ecosystem and green innovation capability partially mediate the relationship between scientific leadership and sustainable innovation. Additionally, environmental dynamism moderates the relationship between green innovation capability and sustainable innovation, such that the relationship is stronger in highly dynamic environments. These findings contribute to the leadership and innovation literature by elucidating the micro-foundations of sustainable innovation and highlighting the critical role of scientific leadership in fostering organizational capabilities for environmental sustainability.

## Introduction

The escalating global environmental crisis has precipitated an urgent imperative for organizations to transition toward sustainable business models (Fernando et al., 2019). Knowledge-intensive firms (KIFs), characterized by their reliance on specialized knowledge and intellectual capital, occupy a pivotal position in driving sustainable innovation (Aslesen and Isaksen, 2007). These organizations leverage their cognitive resources to develop environmentally benign technologies and processes that reconcile economic objectives with ecological stewardship. However, the mechanisms through which leadership shapes sustainable innovation capabilities in KIFs remain inadequately understood.

Leadership scholarship has increasingly recognized the distinctive challenges of guiding scientifically oriented organizations. Scientific leadership, conceptualized as the capacity to articulate compelling scientific visions, foster intellectual curiosity, and orchestrate knowledge creation processes, represents a critical yet underexplored leadership paradigm (Vlok, 2012). Unlike conventional leadership approaches that prioritize administrative efficiency and hierarchical control, scientific leadership emphasizes epistemic authority, methodological rigor, and the cultivation of exploratory learning environments (Hemlin et al., 2014).

The dynamic capabilities framework posits that sustained competitive advantage emanates from organizations’ ability to integrate, build, and reconfigure internal and external competencies in response to rapidly changing environments (Teece and Shuen, 1997). From this theoretical vantage point, sustainable innovation represents not merely an outcome of fortuitous technological discoveries but rather the manifestation of deliberately cultivated organizational capabilities (Bari et al., 2022). Specifically, talent ecosystem and green innovation capability constitute two interrelated dynamic capabilities that enable KIFs to translate scientific leadership into tangible sustainable innovation outcomes. In view of this, this study takes knowledge intensive enterprises in China as the background, aiming to explore the impact of scientific leadership on sustainable innovation, as well as the mediating role and boundary conditions of talent ecosystem and green innovation capability.

This study makes several contributions to the extant literature. First, it extend leadership theory by articulating the concept of scientific leadership and explicating its distinctive characteristics in knowledge-intensive contexts. Second, it advance the sustainable innovation literature by identifying talent ecosystem and green innovation capability as critical mediating mechanisms that translate leadership influence into environmental performance. Third, it contribute to the dynamic capabilities literature by examining how environmental dynamism moderates the capability-performance relationship, thereby elucidating the boundary conditions of our theoretical model.

## Theoretical background and hypotheses

### Scientific leadership

Scientific leadership represents a specialized form of intellectual leadership that emerges in contexts where knowledge creation and scientific discovery constitute primary organizational objectives. Drawing upon the foundational work of Vlok (2012), it conceptualize scientific leadership as comprising three interconnected dimensions: epistemic vision, methodological stewardship, and intellectual community building. Epistemic vision refers to the leader’s capacity to articulate compelling research agendas that address significant scientific and societal challenges. Methodological stewardship encompasses the leader’s commitment to rigorous research methodologies and evidence-based decision-making. Intellectual community building involves creating organizational contexts that foster intellectual exchange, constructive critique, and collaborative knowledge creation.

The theoretical foundations of scientific leadership can be traced to the broader literature on knowledge-oriented leadership (Sadeghi and Rad, 2018) and creative leadership in scientific contexts (Hemlin et al., 2014). Unlike transformational leadership, which emphasizes charismatic influence and inspirational motivation, scientific leadership derives its legitimacy from epistemic authority and demonstrated scientific competence (Mabey et al., 2012). This form of leadership is particularly salient in KIFs, where organizational success depends critically on the ability to attract, develop, and retain scientific talent.

### Talent ecosystem

The concept of talent ecosystem extends traditional talent management perspectives by emphasizing the interconnected, dynamic, and co-evolutionary nature of talent-related organizational processes. Rather than conceptualizing talent as a static stock of human capital, the ecosystem perspective views talent as an emergent property of complex interactions among individuals, teams, and organizational systems (Rožman et al., 2023). A robust talent ecosystem encompasses three critical components: talent attraction mechanisms that draw high-potential individuals into the organization, talent development practices that enhance individual and collective capabilities, and talent retention strategies that sustain organizational knowledge stocks.

From a dynamic capabilities perspective, talent ecosystem represents a higher-order capability that enables organizations to continuously renew their human capital resources in alignment with strategic objectives (Ahmed et al., 2021). In knowledge-intensive contexts, the quality of the talent ecosystem directly influences the organization’s capacity for innovation and adaptation (Ingram, 2016). Scientific leadership, with its emphasis on intellectual community building and epistemic vision, is hypothesized to positively influence the development and maintenance of robust talent ecosystems.

### Green innovation capability

Green innovation capability refers to the organizational capacity to develop and implement environmentally sustainable products, processes, and services that reduce ecological impact while supporting competitive positioning (Albort-Morant et al., 2016). This capability encompasses green product innovation, which involves developing environmentally friendly products; green process innovation, which focuses on reducing environmental impacts in production processes; and green managerial innovation, which entails implementing environmentally oriented organizational practices.

Contemporary research increasingly frames green innovation not merely as a technological outcome but as a dynamic organizational capability that emerges when firms systematically cultivate employees’ environmental knowledge, creativity, and sustainability-oriented behaviors (Akhtar et al., 2024). Within this perspective, green innovation capability functions as a catalyst and enabler of sustainable innovation, translating human capital and organizational resources into measurable environmental outcomes (Frempong et al., 2021).

### Sustainable innovation

Sustainable innovation represents the organizational capacity to develop and implement innovations that simultaneously address economic, environmental, and social objectives (Fernando et al., 2019). Unlike traditional innovation paradigms that prioritize financial performance, sustainable innovation emphasizes the integration of environmental and social considerations into the innovation process. This conceptualization aligns with the triple bottom line framework, which posits that organizational success should be evaluated across economic, ecological, and social dimensions.

In the context of KIFs, sustainable innovation assumes particular significance given these organizations’ potential to develop knowledge-intensive solutions to environmental challenges (Tödtling et al., 2006). The development of sustainable innovation capabilities requires not only technical expertise but also organizational processes that facilitate the integration of diverse knowledge domains and stakeholder perspectives (Jenssen and Nybakk, 2013).

### Hypotheses development

#### Scientific leadership and talent ecosystem

Scientific leadership is hypothesized to positively influence the development of robust talent ecosystems in KIFs. Leaders who articulate compelling epistemic visions create a sense of intellectual purpose that attracts high-quality scientific talent. Furthermore, scientific leaders who demonstrate methodological stewardship establish organizational norms that value intellectual rigor and evidence-based reasoning, thereby creating developmental contexts that enhance individual capabilities (Vlok, 2012). Finally, scientific leaders who prioritize intellectual community building foster collaborative environments that promote knowledge sharing and collective learning (Hemlin et al., 2014).

The relationship between scientific leadership and talent ecosystem is further reinforced by the signaling function of leadership behavior. In knowledge-intensive labor markets, prospective employees evaluate organizational attractiveness based on cues regarding intellectual climate and developmental opportunities (Mabey et al., 2012). Scientific leadership signals an organization-wide commitment to intellectual excellence, thereby enhancing the firm’s ability to attract and retain high-caliber talent. Based on this reasoning, it proposes:

*Hypothesis 1a:* Scientific leadership is positively related to talent ecosystem quality in knowledge-intensive firms.

#### Scientific leadership and green innovation capability

Scientific leadership is also hypothesized to enhance green innovation capability. Leaders who articulate epistemic visions that incorporate environmental sustainability create organizational agendas that prioritize green innovation objectives (Bari et al., 2022). Methodological stewardship, when applied to environmental challenges, establishes rigorous approaches to evaluating and improving environmental performance (Albort-Morant et al., 2016). Moreover, intellectual community building facilitates the cross-functional collaboration necessary for developing integrated green innovation solutions (Akhtar et al., 2024).

The influence of scientific leadership on green innovation capability is mediated through organizational learning processes. Scientific leaders create organizational contexts that encourage experimentation, tolerate failure, and promote knowledge accumulation from environmental initiatives. These learning-oriented contexts enable the development of specialized knowledge and routines that constitute green innovation capability. Accordingly, it proposes:

*Hypothesis 1b:* Scientific leadership is positively related to green innovation capability in knowledge-intensive firms.

#### Talent ecosystem and sustainable innovation

A robust talent ecosystem provides the human capital foundation necessary for sustainable innovation. High-quality talent ecosystems ensure that organizations possess the diverse knowledge stocks required to address complex sustainability challenges (Aman-Ullah et al., 2022). Furthermore, well-developed talent ecosystems facilitate knowledge integration across disciplinary boundaries, enabling the synthesis of technical, environmental, and market knowledge necessary for successful sustainable innovation (AlQershi et al., 2021).

The relationship between talent ecosystem and sustainable innovation is reinforced by the motivational mechanisms inherent in developmental organizational contexts. When employees perceive that their organization invests in their professional growth and provides opportunities for meaningful work, they exhibit higher levels of engagement and creative contribution (Costa et al., 2023). This enhanced engagement translates into greater innovative effort directed toward sustainability objectives. Thus, it proposes:

*Hypothesis 2a:* Talent ecosystem is positively related to sustainable innovation in knowledge-intensive firms.

#### Green innovation capability and sustainable innovation

Green innovation capability directly enables sustainable innovation by providing the organizational processes and routines necessary to develop and implement environmentally beneficial innovations. Organizations with well-developed green innovation capabilities possess the technical knowledge to identify environmental improvement opportunities, the managerial systems to prioritize and resource green initiatives, and the implementation capabilities to translate green concepts into marketable products and processes (Frempong et al., 2021).

The relationship between green innovation capability and sustainable innovation is strengthened by the cumulative nature of capability development. As organizations accumulate experience with green innovation initiatives, they develop specialized knowledge stocks and routines that facilitate subsequent innovation efforts. This capability accumulation creates a virtuous cycle in which green innovation capability and sustainable innovation outcomes mutually reinforce each other over time. Based on this reasoning, it proposes:

*Hypothesis 2b:* Green innovation capability is positively related to sustainable innovation in knowledge-intensive firms.

#### Mediating role of talent ecosystem, green innovation capability on scientific leadership and sustainable innovation

It further proposes that talent ecosystem and green innovation capability mediate the relationship between scientific leadership and sustainable innovation. Scientific leadership influences sustainable innovation not only through direct mechanisms but also indirectly through its effects on organizational capabilities (Cortes and Herrmann, 2021). Specifically, scientific leadership enhances talent ecosystem quality, which in turn provides the human capital foundation for sustainable innovation. Similarly, scientific leadership develops green innovation capability, which enables the organizational processes necessary for translating sustainability aspirations into tangible outcomes.

The mediating role of these capabilities is consistent with the dynamic capabilities perspective, which posits that leadership influences organizational outcomes primarily through its effects on capability development (Teece, 2010). Rather than directly determining innovation outcomes, leadership creates organizational contexts that enable the development and deployment of capabilities that subsequently drive performance (Hitt and Duane, 2002). This indirect influence pathway suggests:

*Hypothesis 3:* Talent ecosystem and green innovation capability mediate the relationship between scientific leadership and sustainable innovation.

#### Moderating role of environmental dynamism

Environmental dynamism refers to the rate and unpredictability of change in an organization’s external environment (Dess and Beard, 1984). In highly dynamic environments characterized by rapid technological change, shifting regulatory requirements, and evolving customer preferences, organizational capabilities assume heightened strategic significance. It hypothesize that environmental dynamism moderates the relationship between green innovation capability and sustainable innovation.

Research has shown that green innovation capability is the core lever for small and medium-sized enterprises to achieve sustainable innovation in resource constrained situations, and the construction of this capability requires both internal knowledge management support and multi-party collaboration in an open innovation ecosystem (Achmad et al., 2023; Achmad and Wiratmadja, 2024; Achmad and Wiratmadja, 2025). Continuous innovation is no longer just the continuation of technological research and development, but a strategic process in which organizations continuously adapt, integrate, and restructure internal and external resources through dynamic capabilities, achieving a balance between economic performance and environmental sustainability.

In dynamic environments, organizations face intensified pressure to continuously adapt their environmental strategies in response to emerging challenges and opportunities (Breznik and Lahovnik, 2016). Green innovation capability enables organizations to rapidly reconfigure their environmental practices in alignment with changing external conditions (Vanpoucke et al., 2014). Conversely, in stable environments, the returns to green innovation capability may be diminished as organizations can rely on established environmental practices without facing significant adaptation pressures. Thus, it proposes:

*Hypothesis 4:* Environmental dynamism moderates the relationship between green innovation capability and sustainable innovation, such that the relationship is stronger when environmental dynamism is high.

**Figure 1:**
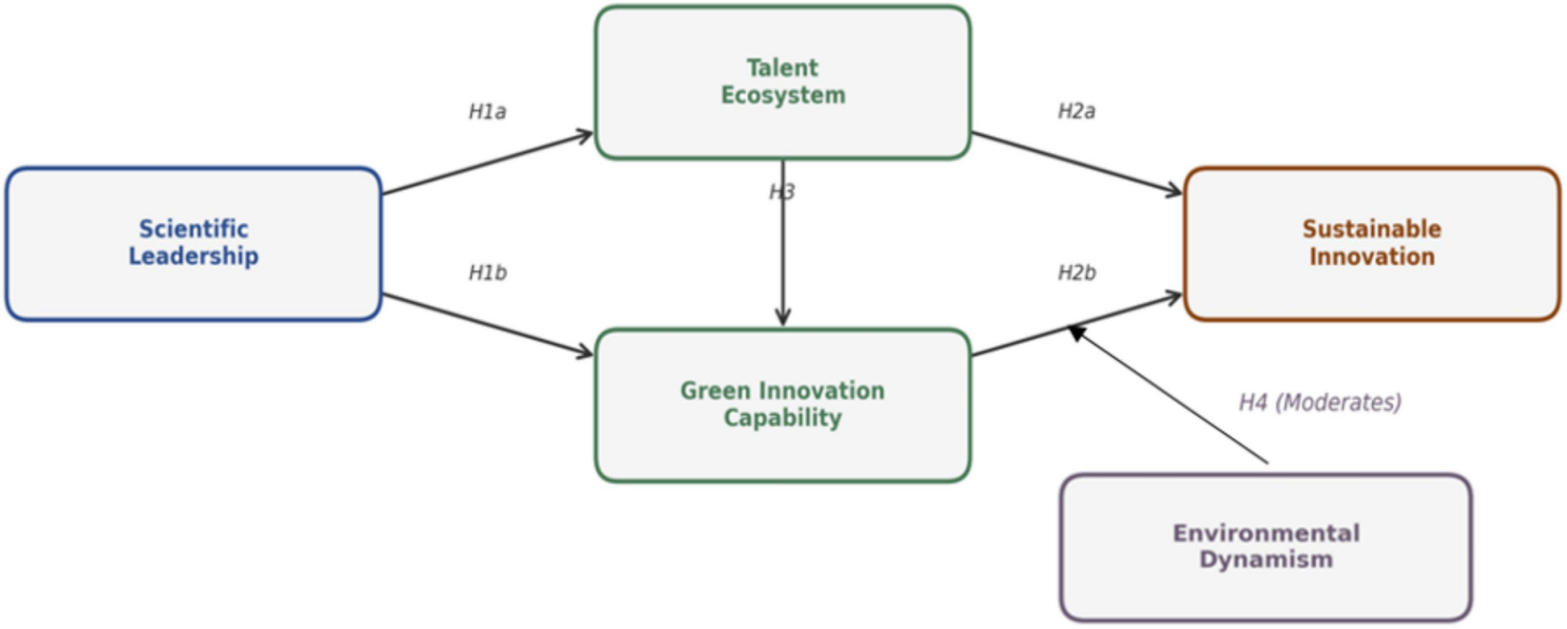
Research framework.

In conclusion, Figure 1 presents the research framework.

## Methodology

### Sample and data collection

It tested these hypotheses using survey data collected from knowledge-intensive firms operating in China’s high-technology sectors, including information technology, biotechnology, new energy, and advanced manufacturing industries. These sectors were selected because they represent contexts where scientific leadership and sustainable innovation are particularly salient. The sampling frame consisted of firms listed in the China High-Tech Enterprise Directory and the National Enterprise Credit Information Publicity System.

Data collection proceeded in two waves to reduce common method bias. In the first wave (January 2024), it contacted senior executives (CEOs, general managers, or R&D directors) to complete questionnaires assessing scientific leadership, environmental dynamism, and control variables. In the second wave (April 2024), it contacted different informants (HR directors or sustainability managers) from the same firms to provide ratings of talent ecosystem, green innovation capability, and sustainable innovation. This temporal separation and informant separation strategy helps mitigate concerns regarding common method variance.

It distributed questionnaires to 650 firms and received 312 responses in the first wave (48.0% response rate). After matching with second-wave responses and excluding cases with missing data, the final sample consisted of 287 firms, representing a 44.2% effective response rate. Non-response bias was assessed by comparing early and late respondents on key demographic variables; no significant differences were found, suggesting that non-response bias is unlikely to threaten the validity of our findings. Table 1 presents the sample characteristics.

**Table 1:** Sample characteristics (*N* = 287).

Characteristic	Frequency	Percentage
Industry type		
Information technology	98	34.1%
Biotechnology	67	23.3%
New energy	62	21.7%
Advanced manufacturing	60	20.9%
Firm size (employees)		
Mean (SD)	287 (156)	
Range	50–1,200	
Firm age (years)		
Mean (SD)	12.4 (6.8)	
Range	3–28	
R&D intensity (%)		
Mean (SD)	8.7 (4.2)	

### Measures

#### Scientific leadership

Scientific leadership was measured using a 12-item scale adapted from Vlok (2012). The scale captures three dimensions: epistemic vision (4 items, e.g., “Our top leader articulates a compelling vision for scientific excellence”), methodological stewardship (4 items, e.g., “Our top leader emphasizes rigorous evidence-based decision making”), and intellectual community building (4 items, e.g., “Our top leader creates opportunities for collaborative knowledge sharing”). Respondents rated items on a 7-point Likert scale (1 = strongly disagree, 7 = strongly agree). Cronbach’s alpha for this scale was 0.91.

#### Talent ecosystem

Talent ecosystem was measured using a 9-item scale developed based Rožman et al. (2023). The scale assesses three components: talent attraction (3 items, e.g., “Our firm successfully attracts high-quality scientific talent”), talent development (3 items, e.g., “Our firm provides excellent opportunities for professional growth”), and talent retention (3 items, e.g., “Our firm effectively retains key scientific personnel”). Responses were provided on a 7-point Likert scale. Cronbach’s alpha was 0.88.

#### Green innovation capability

Green innovation capability was measured using an 8-item scale adapted from Albort-Morant et al. (2016). The scale captures green product innovation capability (3 items), green process innovation capability (3 items), and green managerial innovation capability (2 items). Sample items include “Our firm is capable of developing environmentally friendly products” and “Our firm effectively implements environmentally sustainable production processes.” Cronbach’s alpha was 0.89.

#### Sustainable innovation

Sustainable innovation was measured using a 10-item scale based on Fernando et al. (2019) and Frempong et al. (2021). The scale assesses the extent to which the firm has developed innovations that address environmental objectives, including eco-efficiency improvements, pollution reduction, and resource conservation. Sample items include “Our firm has developed innovations that significantly reduce environmental impact” and “Our firm consistently introduces innovations that improve ecological sustainability.” Cronbach’s alpha was 0.92.

#### Environmental dynamism

Environmental dynamism was measured using a 5-item scale adapted from Dess and Beard (1984). The scale captures the rate of change and unpredictability in the firm’s competitive environment, including technological change, customer preference shifts, and competitive intensity. Sample items include “The technology in our industry changes rapidly” and “Customer preferences in our industry are highly unpredictable.” Cronbach’s alpha was 0.85.

#### Control variables

It controlled for several firm-level characteristics that may influence sustainable innovation: firm size (measured as the natural logarithm of total employees), firm age (measured as years since establishment), industry type (dummy variables for IT, biotechnology, new energy, and advanced manufacturing), and R&D intensity (measured as R&D expenditure divided by total revenue).

## Results

### Measurement model

It conducted confirmatory factor analysis (CFA) to assess the discriminant validity of our key constructs (see Table 2). The five-factor model (scientific leadership, talent ecosystem, green innovation capability, sustainable innovation, and environmental dynamism) demonstrated acceptable fit (chi-square/df = 2.14, CFI = 0.94, TLI = 0.93, RMSEA = 0.063, SRMR = 0.058). This model fit significantly better than alternative nested models, supporting the discriminant validity of our measures.

**Table 2:** Confirmatory factor analysis results.

Model	Chi-square/df	CFI	RMSEA
Five-factor model (hypothesized)	2.14	0.94	0.063
Four-factor model (SL + TE)	4.87	0.82	0.118
Four-factor model (GIC + SI)	4.52	0.84	0.112
Single-factor model	8.23	0.52	0.182

SL = Scientific Leadership; TE = Talent Ecosystem; GIC = Green Innovation Capability; SI = Sustainable Innovation. All chi-square differences between the hypothesized model and alternative models are significant at *p* < 0.001.

It also examined the potential threat of common method bias using Harman’s single-factor test and the marker variable technique. The single-factor model showed poor fit (CFI = 0.52, RMSEA = 0.182), suggesting that common method bias is not a serious concern. Furthermore, the inclusion of a marker variable did not substantially change the significance or magnitude of our hypothesized relationships.

### Descriptive statistics

Descriptive statistics of variables show that: Scientific leadership was positively correlated with talent ecosystem (r = 0.52, *p* < 0.01), green innovation capability (r = 0.48, *p* < 0.01), and sustainable innovation (r = 0.45, *p* < 0.01). Talent ecosystem and green innovation capability were both positively correlated with sustainable innovation (r = 0.56, *p* < 0.01 and r = 0.61, *p* < 0.01). These correlations provide preliminary support for our hypothesized relationships.

### Hypothesis testing

It tested our hypotheses using hierarchical multiple regression analysis and bootstrapped mediation analysis. Table 3 presents the regression results for the direct and mediating effects.

**Table 3:** Regression results for hypothesis testing.

Variable	Model 1 TE	Model 2 TE	Model 3 GIC	Model 4 GIC	Model 5 SI	Model 6 SI	Model 7 SI
Control variables							
Firm size	0.08	0.06	0.09	0.07	0.11*	0.08	0.08
Firm age	−0.05	−0.04	−0.03	−0.02	−0.04	−0.03	−0.03
R&D intensity	0.12*	0.10	0.15**	0.13*	0.18**	0.14*	0.14*
Independent variable							
Scientific leadership		0.48***		0.42***	0.38***	0.18**	0.18**
Mediators							
Talent ecosystem						0.35***	0.35***
Green innovation capability						0.41***	0.40***
Moderator							
Environmental dynamism (ED)						0.12*	0.11*
Interaction							
GIC x ED							0.14**
R-squared	0.03	0.26	0.04	0.22	0.18	0.47	0.49
Adjusted R-squared	0.02	0.25	0.03	0.21	0.17	0.46	0.48
F-statistic	2.87	32.14***	3.82	26.48***	20.56***	46.28***	42.15***

*N* = 287. TE = Talent Ecosystem; GIC = Green Innovation Capability; SI = Sustainable Innovation. Standardized beta coefficients are reported. * *p* < 0.05, ** *p* < 0.01, *** *p* < 0.001.

Hypothesis 1a proposed that scientific leadership is positively related to talent ecosystem. As shown in Model 2 of Table 3, scientific leadership had a significant positive effect on talent ecosystem (beta = 0.48, *p* < 0.001), supporting Hypothesis1a. Hypothesis 1b posited that scientific leadership is positively related to green innovation capability. Model 4 shows that scientific leadership had a significant positive effect on green innovation capability (beta = 0.42, *p* < 0.001), supporting Hypothesis 1b.

Hypothesis 2a predicted that talent ecosystem is positively related to sustainable innovation. Model 6 indicates that talent ecosystem had a significant positive effect on sustainable innovation (beta = 0.35, *p* < 0.001), supporting Hypothesis 2a. Hypothesis 2b proposed that green innovation capability is positively related to sustainable innovation. Model 6 shows that green innovation capability had a significant positive effect on sustainable innovation (beta = 0.41, *p* < 0.001), supporting Hypothesis 2b.

Hypothesis 3 proposed that talent ecosystem and green innovation capability mediate the relationship between scientific leadership and sustainable innovation. It tested this mediation hypothesis using bootstrapped confidence intervals based on 5,000 resamples. The indirect effect of scientific leadership on sustainable innovation through talent ecosystem was significant (indirect effect = 0.12, 95% CI [0.07, 0.18]), as was the indirect effect through green innovation capability (indirect effect = 0.15, 95% CI [0.09, 0.22]). The direct effect of scientific leadership on sustainable innovation remained significant after including the mediators (beta = 0.18, *p* < 0.01), indicating partial mediation. These results support Hypothesis 3.

Hypothesis 4 proposed that environmental dynamism moderates the relationship between green innovation capability and sustainable innovation. It tested this moderation hypothesis using hierarchical regression with interaction terms. As shown in Model 7 of Table 3, the interaction between green innovation capability and environmental dynamism was significant and positive (beta = 0.14, *p* < 0.01), supporting Hypothesis 4. Figure 1 illustrates this moderating effect, showing that the relationship between green innovation capability and sustainable innovation is stronger at high levels of environmental dynamism.

## Discussion

### Theoretical implications

This study makes several contributions to the leadership and innovation literature. First, it extends leadership theory by articulating the concept of scientific leadership and demonstrating its distinctive effects on organizational capabilities in knowledge-intensive contexts. While prior research has examined various leadership styles in innovation settings, scientific leadership represents a unique configuration that emphasizes epistemic authority, methodological rigor, and intellectual community building. Our findings suggest that this leadership approach is particularly effective in fostering the organizational capabilities necessary for sustainable innovation.

Second, it contributes to the sustainable innovation literature by identifying talent ecosystem and green innovation capability as critical mediating mechanisms. Prior research has often treated sustainable innovation as a direct outcome of leadership or strategy, without adequately specifying the organizational processes through which leadership influence is transmitted. Our findings demonstrate that scientific leadership shapes sustainable innovation primarily through its effects on organizational capabilities, highlighting the importance of capability development in the sustainability transformation process.

Third, it advances the dynamic capabilities literature by examining how environmental dynamism moderates the capability-performance relationship. Our findings suggest that the returns to green innovation capability are contingent on environmental conditions, with stronger effects observed in highly dynamic environments. This contingency perspective enriches our understanding of when and why dynamic capabilities create competitive advantage. Achmad and Wiratmadja (2025) found that: encourage small and medium-sized business owners and managers to integrate green innovation into their business strategies, while strengthening internal capabilities to support sustainable practices. By doing so, Batik’s small and medium-sized enterprises can improve organizational performance, maintain market competitiveness, and achieve long-term sustainable development.

### Practical implications

Our findings offer several practical implications for managers of knowledge-intensive firms. First, leaders should recognize the importance of developing scientific leadership capabilities, particularly in contexts where sustainable innovation is a strategic priority. This involves cultivating epistemic vision, demonstrating methodological stewardship, and building intellectual communities that foster collaborative knowledge creation.

Second, managers should invest in developing robust talent ecosystems that attract, develop, and retain high-quality scientific talent. Our findings suggest that talent ecosystem quality is a critical pathway through which leadership influences sustainable innovation outcomes. This implies that sustainability-focused leadership must be accompanied by human resource practices that support talent development.

Third, organizations should prioritize the development of green innovation capabilities, particularly in dynamic environments. Our findings indicate that green innovation capability is especially valuable when external conditions are rapidly changing, suggesting that organizations should invest in building flexible environmental management systems that enable rapid adaptation.

### Limitations and future research

This study has several limitations that suggest directions for future research. First, our cross-sectional design limits our ability to make strong causal inferences. Future research should employ longitudinal designs to better establish the temporal sequence of relationships among scientific leadership, organizational capabilities, and sustainable innovation.

Second, our sample was drawn from Chinese KIFs, which may limit the generalizability of our findings to other national and organizational contexts. Future research should examine whether our theoretical model holds in different cultural and institutional settings.

Third, it focused on two mediating mechanisms (talent ecosystem and green innovation capability) but acknowledge that other mechanisms may also be at play. Future research could explore additional pathways, such as organizational learning, knowledge sharing, and stakeholder engagement.

Finally, our measure of sustainable innovation focused primarily on environmental outcomes. Future research could extend our model to incorporate social sustainability dimensions, providing a more comprehensive assessment of sustainable innovation performance.

## Conclusion

This study advances our understanding of how scientific leadership shapes sustainable innovation in knowledge-intensive firms. By identifying talent ecosystem and green innovation capability as critical mediating mechanisms, it illuminate the organizational processes through which leadership influence is transmitted to innovation outcomes. Furthermore, our findings regarding the moderating role of environmental dynamism highlight the contingent nature of capability-performance relationships. These insights contribute to both theoretical development and practical guidance for organizations seeking to enhance their sustainable innovation capabilities.

## Data Availability

The original contributions presented in the study are included in the article/supplementary material, further inquiries can be directed to the corresponding author.
